# Psychometric Properties of the Self-as-Context Scale with a University Counseling Center Sample

**DOI:** 10.3390/bs15070910

**Published:** 2025-07-04

**Authors:** Robert D. Zettle, Grace A. Lyons, Jonathan M. Larson, Christopher Leonard

**Affiliations:** 1 Department of Psychology, Wichita State University, Wichita, KS 67260, USA; galyons@shockers.wichita.edu (G.A.L.); jmlarson1@shockers.wichita.edu (J.M.L.); 2 Counseling and Psychological Services, Wichita State University, Wichita, KS 67260, USA; christopher.leonard@wichita.edu

**Keywords:** self-as-context, psychometric properties, measurement invariance, known-groups validity

## Abstract

The model upon which acceptance and commitment therapy is based posits that its outcomes are mediated by increased psychological flexibility as a core process. Of the six subprocesses contributing to psychological flexibility, self-as-context has been investigated the least due to a lack of adequate assessment. An evaluation of the psychometric properties of at least one such measure—the Self-as-Context Scale (SACS)—has been primarily limited to nonclinical populations. To address this omission, we administered the SACS to students (*N* = 132) seeking psychological services from their university counseling center. A confirmatory factor analysis failed to find an adequate fit for a previously reported two-dimensional model of the SACS, suggesting that only total scores may be appropriate in research and practice involving clinical samples. All 10 items satisfactorily loaded on a single factor to produce reliable total scaled scores, which were, as expected, significantly lower for our participants than those from a general college student sample. Even lower scores were obtained for outpatients of a psychology training clinic compared to our sample, which provided additional support for the known-groups validity of the SACS. The limitations of the findings and implications for further investigations of the measure’s psychometric and functional properties are discussed.

## 1. Introduction

Over the last two to three decades, a number of distinct, yet related, psychotherapeutic approaches have emerged that have been collectively recognized as representing a third wave of cognitive–behavioral therapy (CBT; Hayes, 2004). Approaches within this new generation of CBT include acceptance and commitment therapy (ACT; Hayes et al., 2012), dialectical behavior therapy (Linehan, 1993), functional analytic psychotherapy (Kohlenberg & Tsai, 1991), integrative behavioral couples therapy (Christensen et al., 2020), and mindfulness-based cognitive therapy (Segal et al., 2018), among others. While these associated approaches may differ somewhat in their specific therapeutic techniques and components, as well as in the particular presenting issues and concerns they address, they generally share indirect change strategies, such as the application of acceptance and mindfulness, in which altering how clients relate and respond to unwanted psychological experiences and emotional states, such as depression and anxiety, is emphasized over attaining symptomatic relief from them.

As a transdiagnostic approach applied to diverse forms of human suffering, ACT appears to have been more extensively investigated than most of its fellow third-wave forms of CBT, as reflected by an ever-expanding body of both outcome and process research (Levin et al., 2024). At the time of this writing, there have been nearly 1350 randomized controlled trials of ACT that have led to its recognition as an evidence-based intervention for a range of both behavioral and physical health concerns (Association for Contextual Behavioral Science, n.d.). For example, the World Health Organization (2020) acknowledges ACT within its guidelines as being empirically supported for reducing chronic pain in children and adolescents. The Society of Clinical Psychology, Division 12 of the American Psychological Association, also recognizes ACT as having strong empirical support in the treatment of chronic pain (Society of Clinical Psychology, n.d.-a), as well as modest support in addressing depression (Society of Clinical Psychology, n.d.-b), mixed anxiety (Society of Clinical Psychology, n.d.-c), obsessive–compulsive disorder (Society of Clinical Psychology, n.d.-d), and psychosis (Society of Clinical Psychology, n.d.-e).

Beginning with the earliest outcome studies (Zettle & Hayes, 1986; Zettle & Rains, 1989), researchers of ACT have not only been interested in evaluating its relative efficacy, but also in investigating conceptually meaningful variables that may moderate and/or mediate therapeutic change (Zettle & Wilson, 2023). At a process level, the objective of ACT is to increase psychological flexibility, or the ability to persist in actions congruent with personal values in the face of unwanted thoughts, feelings, bodily sensations, and other private events that might serve as barriers to such behavior (Hayes et al., 2012). The following six subprocesses are, in turn, posited to collectively, as well as independently, contribute to psychological flexibility within a conceptual model often referred to as the “hexaflex” (Hayes et al., 2012, p. 62): (a) acceptance or an openness to remain in contact with unwanted psychological experiences, (b) defusion or disentangling from unhelpful thoughts and other verbal constructions, (c) present moment awareness of flexibly attending to experiences in the here-and-now, (d) self-as-context as a type of disengaged perspective taking, (e) values as meaningful life directions, and (f) committed action as behavioral patterns congruent with one’s chosen values.

Enough process research has been conducted in relation to ACT that several systematic reviews and meta-analyses of such investigations have been published within the last 5 years (e.g., Akbari et al., 2022; Gloster et al., 2020; Johannsen et al., 2022; Tunç et al., 2023). However, the degree to which meta-analytic findings have been seen as providing sufficient evidence that increased psychological flexibility, as well as the separate subprocesses that contribute to it, mediate treatment outcomes in ACT has been the focus of some debate. While Levin et al. (2024) concluded that the supportive evidence for increased psychological flexibility as a mechanism of action in ACT is adequate, others have argued that measures of the construct, as well as its subprocesses, appear to be psychometrically weak and lack sufficient sensitivity and specificity (Arch et al., 2023).

One point on which both parties seem to be in agreement within the debate about the status of mediational research within ACT is that self-as-context has been understudied as a purported separate subprocess of psychological flexibility (Arch et al., 2023; Fishbein et al., 2020). To a considerable degree, until relatively recently, this neglect appears to have resulted from the absence of acceptable measures of the process (Godbee & Kangas, 2020), which, in turn, may, at least in part, be attributable to the somewhat ephemeral nature of self-as-context and challenges in defining and assessing it. For the purpose of this paper, what follows is an overview of the purported development of self-as-context and several of its defining features. Interested readers are advised to consult the work of McHugh and Stewart (2012) for a more detailed coverage of these matters.

Also frequently referred to as the “contextual”, as well as “observing self” (Hayes et al., 1999, p. 184), self-as-context is thought to develop fairly early in life from inquiries directed to children from members of their larger verbal–social community about what they have done, what they are doing, and what they will do (Skinner, 1974). What ultimately emerges from such interchanges is a repertoire of self-awareness, or “seeing that I am seeing,” that occurs from a unique and invariant perspective, which has been likened to a transcendent sense of spirituality (Hayes, 1984). Unwanted private events, such as distressing thoughts and emotions, observed in this distanced manner may be experienced as distinct from who we are, as defined by our self-concept (Yu et al., 2016). The clinical implications are that such a perspective on unwanted psychological events presumably facilitates acceptance rather than entangled and counterproductive efforts to control them (Hayes et al., 2012, pp. 88–90).

In effect, from the vantage point of the contextual or observing self, I am both bigger than and distinct from my thoughts and feelings, even when they are specifically about me (e.g., “I’m a loser.”). Moreover, I am not defined by them. More simply put, I have thoughts, *and* I am not my thoughts. The following sky and the weather metaphor is frequently used within ACT to help clients distinguish between unwanted psychological experiences and their sense of a contextual self: “Your observing self is like the sky. Thoughts and feelings are like the weather. The weather changes continually, but no matter how bad it gets, it can’t harm the sky in any way” (Harris, 2009, p. 175).

The historical lack of research on self-as-context, particularly relative to that of other hexaflex processes such as acceptance and defusion, has at least modestly increased within the last decade with the development of five different self-report measures of the contextual self and related dimensions—the Reno Inventory of Self-Perspective (RISP; Jeffcoat, 2015), the Self-Experiences Questionnaire (SEQ; Yu et al., 2016), the Self-as-Context Scale (SACS; Zettle et al., 2018), the self-as-context subscale of the Multidimensional Psychological Flexibility Inventory (MPFI; Rolffs et al., 2018), and the Questionnaire on Self-Transcendence (QUEST; Fishbein et al., 2020). While all five instruments have, thus far, been shown to have acceptable psychometric properties (Fang et al., 2022, 2023; Peixoto et al., 2019; Yu et al., 2017), their application to clinical samples has, unfortunately, been fairly limited. For example, other than the inclusion of a modest number of community adults seeking services from an outpatient psychology training clinic in its development (i.e., Sample 9 of Zettle et al., 2018), the psychometric properties of the SACS with clinical populations have not been further evaluated. Accordingly, knowing more about these matters could better inform the use of SACS in the clinical research and practice of ACT in particular, as well as of related third-wave forms of CBT more generally.

The major purpose of this project was to, at least partially, address the lack of research on the psychometric properties of the SACS with clinical samples by assessing the internal reliability measurement invariance of the SACS administered to a primary sample of students requesting psychotherapy from their university counseling center. This also provided an opportunity to further evaluate the known-groups validity of the instrument by comparing scores to a secondary sample of general college students from the same university and the aforementioned group of training clinic outpatients.

## 2. Materials and Methods

### 2.1. Participants

This project was approved by an institutional review board and conducted in accordance with the ethical principles of the American Psychological Association (2017). Beginning in mid-October during the 2022–2023 academic year, students were invited upon intake at our university’s counseling center to complete a brief demographic questionnaire and the SACS. Approximately 90% agreed to participate. The final sample (*N* = 132) was predominantly assigned female at birth (53%), non-Latinx (76%), and White (73%), with a mean age of 21.4 years (see Table 1). Participants self-identified as multiracial (6%), Black (6%), and Asian/Asian American (10%) represented other racial subgroups. As far as could be determined, this primary study sample appeared to be representative of all new student intakes at the counseling center during the time of data collection (*N* = 389) in gender assigned at birth and age. However, this larger sample had significantly lower proportions of both Latinx (12%) and White (59%) students compared to our subsample. Their most common concerns subsequently identified during the delivery of services following intake included anxiety (67%), depression (57%), and stress (48%).

Also presented in Table 1 are demographic data from two other samples against which SACS scores from our primary sample of counseling center clients were compared. The first of these comparison or secondary samples was collected from general students at our university for a recent project evaluating the relative incremental predictive validity of the SACS (Zettle et al., 2025). This general student sample did not differ from our participants in mean age (*M* = 20.7) or in being majority White (80%) but did differ by being more predominantly female based on assignment at birth (70%) and non-Latinx (86%).

The second comparison sample, comprising community adults receiving outpatient psychotherapy through our departmental training clinic (*N* = 54), was collected during the development and initial psychometric evaluation of the SACS (i.e., Sample 9 of Zettle et al., 2018). This group of clients was not surprisingly significantly older than our primary sample (*M* = 38.7) and even more predominantly female (86%).

### 2.2. Self-as-Context Scale (SACS)

The SACS (Zettle et al., 2018) consists of 10 items rated on a 7-point scale (1 = strongly disagree to 7 = strongly agree) that yield a total score when added together. It has demonstrated acceptable levels of temporal and internal consistency (Aydin et al., 2024; Fang et al., 2022; Zettle et al., 2018, 2025), as well as adequate convergent and discriminant validity in nonclinical samples of adolescents (Moran et al., 2018) and college students (Fang et al., 2022; Zettle et al., 2018).

Two subscales—centering and transcending—identified via factor analyses in the original development of the SACS (Zettle et al., 2018) have since been replicated with Chinese college students (Fang et al., 2022). By contrast, a single factor solution emerged with college students from Türkiye as well as with a U.S. sample (*N* = 265) we gathered concurrently at our university for comparison purposes (Aydin et al., 2024). However, an exploratory factor analysis conducted with the same comparison sample of general students included within this study, but collected a year later indicated two dimensions (Aydin et al., 2024). These aggregate findings suggest that the factor structure of the SACS is unstable and somewhat ambiguous. As a result, a recommendation to conduct factor analyses of the SACS to verify if processing data from its separate subscales is warranted in research and clinical practice with differing target samples seems warranted.

The four items of the centering subscale (e.g., “When I am upset, I am able to find a place of calm within myself”) denote relating to unwanted psychological experiences by simply noticing rather than trying to control them. The transcending subscale is assessed with six items (e.g., “As I look back on my life so far, I have a sense that a part of me has been there for all of it”) that reflect the type of invariant perspective-taking encompassed in “seeing that one sees.” A recent comparison of measures related to the contextual self—including the two subscales of the SEQ, the MPFI self-as-context subscale, and the transcending subscale of SACS—found the incremental validity of the centering subscale to be superior in predicting psychological distress and life satisfaction among college students (Zettle et al., 2025).

### 2.3. Data Analysis

We used the structural equation modeling software program Analysis of Moment Structures (AMOS 5.0; Arbuckle, 2003) to conduct a confirmatory factor analysis (CFA) to assess the fit of our primary sample data with the previously reported two-dimensional structure of the SACS (Zettle et al., 2018). A post hoc analysis (Preacher & Coffman, 2006) indicated an acceptable level of power (i.e., 0.82). We considered three measures in evaluating goodness of fit: (a) the normed chi-square (NC), (b) the goodness-of-fit index (GFI; Jöreskog & Sörbom, 1996), and (c) the root mean square error of approximation (RMSEA; Jöreskog & Sörbom, 1996). Insofar as the chi-square statistic (χ^2^) may overestimate the lack of model fit because of its sensitivity to sample size (Bollen, 1989), we divided it by the degrees of freedom (χ^2^/df) to produce a normed chi-square that approaches zero as model fit increases. GFI, by contrast, increases along with model fit, while RMSEA also is indirectly related to model fit. Following the guidelines of Hu and Bentler (1998) and Bollen (1989), we judged NC values of ≤3, GFI values of ≥0.90, and RMSEA values of ≤0.08 as reflective of good model fit.

To further evaluate the known-groups validity of the SACS, we conducted independent *t*-tests using IBM SPSS Statistics (Version 27) to compare scores of our counseling center student clients with those from general student (Zettle et al., 2025) and clinic outpatient samples (Zettle et al., 2018) that had been collected in previous projects.

## 3. Results

### 3.1. Factor Analyses

None of the three fit indices supportive of a bidimensional structure for the SACS were met by our initial CFA. Even after maximizing model fit by allowing errors between pairs of various items to vary, only the guideline for *NC* (2.83) was satisfied; *GFI* = 0.86 and *RMSEA* = 0.12 (0.09–0.15). Consequently, we next completed an exploratory analysis by first using an SPSS syntax program (O’Connor, 2000) to conduct a minimum average partial test (MAP; Velicer, 1976). The revised MAP test (Velicer et al., 2000) revealed a single factor with an eigenvalue of 4.7, accounting for 47% of the variance. All 10 items exhibited acceptable factor loadings (0.49–0.77) and a satisfactory level of internal consistency when compiled into a total score (α = 0.84).

### 3.2. Known-Groups Validity

As anticipated, our participants scored significantly lower on the SACS (*M* = 44.9, *SD* = 10.3) compared to the general student sample (*M* = 49.8, *SD* = 9.4), *t*(445) = 4.87, *p* < 0.001, *d* = 0.50. However, the aforementioned demographic differences between the two samples did not, in turn, appear to account for the expected difference in total SACS scores between them insofar as there were no significant differences in scores based on gender or ethnicity within either sample. As expected, our primary sample reported higher scores than the group of clinic outpatients (*M* = 39.7, *SD* = 10.5), *t*(184) = 3.10, *p* < 0.01, *d* = 0.50.

## 4. Discussion

There appear to be three tentative conclusions that derive from the overall findings of this project, despite its acknowledged limitations. The first, based on our evaluation of the configural invariance of the SACS, is that the two-factor structure initially identified with general samples of both U.S. (Zettle et al., 2018) and Chinese college students (Fang et al., 2022) may not extend to more recent samples of students and other populations. Factor analyses with our primary sample of students seeking mental health services from their university counseling center, as well as those conducted with Turkish college students and at least one of the U.S. samples of Aydin et al. (2024), by contrast, indicated a unidimensional structure. Given such limited configural invariance, it seems appropriate to recommend that the factor structure of the SACS be evaluated specifically for the population to which it is administered if its subscales are to be considered for use in either research or clinical practice.

A second conclusion supported by the results of this project is that total scores on the SACS nonetheless still appear to constitute a reliable scale even if the two subscales are excluded. Continued use of SACS subscales may only be appropriate with certain nonclinical samples, although it would seem advisable to more fully evaluate this matter with participants from differing cultural and linguistic communities, and who more broadly represent more diverse ranges of age, educational levels, and racial/ethnic identities, than U.S. college students.

A third implication of our findings concerns the known-groups validity of the SACS. Total scores significantly differed across the three groups within our comparative analysis in generally predictable ways, consistent with the purported role of the contextual self as a process contributory to psychological flexibility. As expected, our participants reported lower levels of self-as-context than a recent general sample of students at our university (Zettle et al., 2025). This finding moreover seems most attributable to seeking services for psychological distress by our sample, insofar as the results of subsequent analyses dismissed demographic differences between the two groups as a possible explanation.

According to somewhat similar reasoning, the even lower levels of SACS scores for the clinical sample of Zettle et al. (2018) suggests those community adults seeking services from an outpatient clinic were presumably experiencing even greater levels of emotional distress and lower levels of psychological flexibility than participants in our primary sample. However, because this clinical sample was significantly older and more predominantly female, the influence of such demographic variables cannot be ruled out. Accordingly, further research comparing the factor structure of the SACS and scores for clinical and nonclinical samples matched on their demographic profiles is recommended.

## 5. Limitations

A clear methodological limitation of this project is that we opted to not independently evaluate levels of psychological distress in our sample to minimize assessment burden and maximize participation, nor to subsequently seek any related data compiled by the counseling center due to concerns of confidentiality. Further research could address these omissions by more systematically evaluating how the relationship between levels of the contextual self as assessed by SACS total scores and differing forms and degrees of psychological suffering varies both cross-sectionally across nonclinical, subclinical, and clinical samples, as well as longitudinally within each.

Before closing, an ostensible conceptual limitation of this project also seems worthy of some discussion. The practice of judging the quality of psychological measures based on the degree to which they meet the traditional psychometric standards of reliability and validity (American Educational Research Association, 2014) appears to be at least implicitly, if not explicitly, based on a philosophical viewpoint that may be regarded as antithetical to the one on which ACT and the related “hexafex model” are based (Ciarrochi et al., 2016). To the extent this holds, it follows that criteria other than traditional psychometric standards might be legitimately considered as well in more thoroughly evaluating the quality of measures like the SACS that seek to assess the purported subprocesses of psychological flexibility. Our purpose in addressing this matter is not to assert that these alternative evaluative criteria are superior or even preferable to traditional psychometric benchmarks. Rather, it is to argue that their relationship to the assessment of a measure’s psychometric properties be usefully framed as complementary rather than oppositional. Accordingly, from our perspective, a comprehensive qualitative assessment of the SACS in particular, and of measures commonly used in clinical research and practice more generally, should ideally consider evaluative criteria from differing theoretical and philosophical perspectives.

Both classical test theory (Lord & Novick, 1968) and item response theory (Embretson & Reise, 2000) appear to be based on an elementary realistic perspective of the psychological world (Ciarrochi et al., 2016), albeit to somewhat varying degrees. This philosophical viewpoint, which Pepper (1942) referred to as a mechanistic world view, as extended to psychological events and processes essentially assumes that such phenomena naturally exist independently of our scientific efforts to assess and understand them. Accordingly, the quality of measures designed to evaluate psychological variables can, in turn, be meaningfully assessed based on the degree to which they yield consistent data (reliability) and to the extent to which they assess what they purport to (validity) based on a preponderance of the evidence.

The ostensible influence of elemental realism or mechanism is perhaps revealed most clearly in classical test theory’s distinction between true versus observed or obtained scores and the role this conceptualization plays in elevating construct validity to the crown jewel of psychometric standards. Observed assessment data such as SACS total scores ideally should be reflective of participants’ true levels of the contextual self, uncontaminated by measurement error. To the extent that “true scores” are assumed to be relatively stable, test–retest reliability becomes one of several psychometric standards—along with others such as concurrent, discriminant, incremental and known-groups validity—that in the aggregate may provide sufficient evidence for the construct validity of a measure. From this vantage point, there appears to be with the additional results of this study at least further supportive, although far from definitive evidence, for the construct validity of the SACS.

A differential item functioning analysis by Aydin et al. (2024) identified three SACS items that were endorsed at significantly different levels by Turkish and U.S. students. However, as far as we are aware, this rather limited evaluation is the only one to date to assess the psychometric properties of the SACS through the lens of item response theory. While we certainly would recommend and be supportive of such additional research, it does not obviate our concern that item response theory also appears to endorse elemental realism, although seemingly to a less explicit and robust way than critical test theory. Nonetheless, the focus on latent variables as elements that are instrumental in the construction of psychological realities (Borsboom et al., 2003), in our view also contributes to psychometric standards overshadowing other criteria that could at least additionally be meaningfully considered in providing a more comprehensive evaluation of the quality of measures like the SACS. Several of these other benchmarks that involve what is referred to as treatment utility (Hayes et al., 1986, 1987) are suggested by functional contextualism as a philosophy of behavioral science more broadly and its approach to psychological assessment in particular (Ciarrochi et al., 2016).

The interested reader is encouraged to consult other sources that provide more detailed and extensive discussions of the distinctions between elemental realism/mechanism and functional contextualism as alternative philosophies of science (Biglan & Hayes, 2016; Hayes et al., 1988) than that offered here. For our purposes, differing “truth criteria” endorsed by these two perspectives appear to have the most relevant implications for evaluating the quality of psychological measurement. According to Pepper (1942), a truth criterion is the benchmark by which the validity of scientific analyses and purported advancements, such as the development of a new psychological measure or therapeutic approach such as ACT and the model on which it is based, can be assessed. He posits that this evaluative standard for mechanism, or elemental realism, is correspondence-based, while that for various forms of contextualism is “successful working”. A functional contextualistic approach to psychological assessment accordingly is a more explicitly pragmatic one in which measures are viewed as tools whose quality is determined by how well they do their job, rather than by how their data map onto or correspond to psychological theories and models (Ciarrochi et al., 2016). Moreover, the job of psychological assessment is to serve the goal of functional contextualism which is to predict *and* influence behavior with precision, scope, and depth (Biglan & Hayes, 2016).

Recent findings that the centering subscale of the SACS was the only measure of the contextual self to display incremental validity in accounting for variability in both psychological distress and life satisfaction (Zettle et al., 2025) provides at least some modest preliminary support for the assessment quality of SACS from a functional contextualistic perspective. However, it clearly falls short in providing evidence for the treatment utility of the scale or the degree to which it contributes to beneficial therapeutic outcomes (Hayes et al., 1987). This is because meeting certain psychometric standards and demonstrating treatment utility can be independent of each other (Ciarrochi et al., 2016). This orthogonal relationship between the two sets of evaluative criteria can be clarified by briefly considering several research designs commonly used in assessing treatment utility. Interested readers are advised to consult (Hayes et al., 1986, 1987) and Nelson-Gray (2003) for more extensive coverage of them.

Perhaps the simplest means of investigating whether the use of SACS contributes to more efficacious and/or efficient treatment outcomes is to treat its administration as an independent variable within a manipulated assessment design (Ciarrochi et al., 2016). Do clients who complete the measure enjoy better outcomes than their randomly assigned counterparts who do not? Note that an affirmative answer to this question need not be dependent on the psychometric properties of the SACS and that the issue of how the clinician uses the assessment information to provide more effective treatment remains unclear.

To further explore this matter, a somewhat more sophisticated treatment utility design would be required. In a manipulated use of assessment experiment (Nelson-Gray, 2003), all ACT clients might be administered the SACS at pretreatment, but with the assessment information used as the independent variable for only a randomly assigned half of them in determining their program of treatment. Consistent with an increasing emphasis on process-based CBT (Hayes & Hofmann, 2018), clients within this subgroup reporting unusually low levels of the contextual self could receive an enhanced ACT protocol including more experiential exercises and other treatment components designed to facilitate this process. Conversely, those within the same subgroup with relatively high SACS scores, as well as those whose assessment data are not consulted in formulating a treatment plan, could receive a more standard ACT protocol. Superior outcomes for the subgroup whose therapy was informed by their SACS scores would provide supportive evidence of the measure’s treatment utility.

## 6. Conclusions

The results of this project with students seeking psychological services from their university counseling center provide supportive evidence for the known-groups validity of the SACS, while also further documenting its relatively unstable dimensional structure. While its overall psychometric properties warrant the use of total scores in clinical research and practice, we recommend that factor analyses be conducted with “local populations” to verify if the inclusion of SACS subscales is similarly appropriate in such endeavors. We additionally suggest that further item response analyses and treatment utility research be undertaken to more thoroughly evaluate both the psychometric and functional properties of not only the SACS, but also other measures of the contextual self, particularly when used with clinical samples.

## Figures and Tables

**Table 1:** Demographic characteristics and SACS data for primary and comparison samples.

Sample	Counseling Center Students *N* = 132	General Students ^a^ *N* = 315	Clinic Outpatients ^b^ *N* = 54
Age (SD)	21.4 (4.2)	20.7 (3.9)	38.7 (14.4)
Birth Gender			
Male %	47	30	14
Female %	53	70	86
Race			
White %	73	80	72
Non-White %	27	20	28
Ethnicity			
Latinx %	24	14	NA ^c^
Non-Latinx %	76	86	NA
SACS (SD)	44.9 (10.3)	49.8 (9.4)	39.7 (10.5)

^a^ Sample from Zettle et al. (2025) assessment of comparative incremental predictive validity of SACS. ^b^ Sample from Zettle et al. (2018) development of SACS. ^c^ Ethnicity not assessed.

## Data Availability

The data are available upon request from the corresponding author.
